# Increased risk of pelvic fracture after radiotherapy in rectal cancer survivors: A propensity matched study

**DOI:** 10.1002/cam4.2030

**Published:** 2019-05-18

**Authors:** Yu‐Mei Kang, Tze‐Fan Chao, Ti‐Hao Wang, Yu‐Wen Hu

**Affiliations:** ^1^ Division of Radiation Oncology Department of Oncology Taipei Veterans General Hospital Taipei Taiwan; ^2^ Division of Cardiology Department of Medicine Taipei Veterans General Hospital Taipei Taiwan; ^3^ Department of Radiation Oncology China Medical University Hospital Taipei Taiwan; ^4^ School of Medicine National Yang-Ming University Taipei Taiwan

**Keywords:** pelvic fracture, pelvic insufficiency fracture, radiotherapy, rectal cancer

## Abstract

To determine whether radiotherapy (RT) can increase pelvic fracture risk in rectal cancer survivors. Rectal cancer patients who underwent curative surgery between 1996 and 2011 in Taiwan were retrospectively studied using the National Health Insurance Research Database (NHIRD) of Taiwan. ICD‐9 Codes 808, 805.4‐805.7, 806.4‐806.7, and 820 (including pelvic, sacrum, lumbar, and femoral neck fracture) were defined as pelvic fracture. Propensity scores for RT, age, and sex were used to perform one‐to‐one matches between the RT and non‐RT group. Risks of pelvic and arm fractures were compared by multivariable Cox regression. Of the 32 689 patients, 7807 (23.9%) received RT, and 1616 suffered from a pelvic fracture (incidence rate: 1.17/100 person‐years). The median time to pelvic fracture was 2.47 years. After matching, 6952 patients each in the RT and non‐RT groups were analyzed. RT was associated with an increased risk of pelvic fractures in the multivariable Cox model (hazard ratio (HR): 1.246, 95% confidence interval (CI): 1.037‐1.495, *P* = 0.019) but not with arm fractures (HR: 1.013, 95% CI: 0.814‐1.259, *P* = 0.911). Subgroup analyses revealed that RT was associated with a higher pelvic fracture rate in women (HR: 1.431, 95% CI: 1.117‐1.834) but not in men, and the interaction between sex and RT was significant (*P* = 0.03). The HR of pelvic fracture increased 2‐4 years after RT (HR: 1.707, 95% CI: 1.150‐2.534, *P* = 0.008). An increased risk of pelvic fracture is noted in rectal cancer survivors, especially women, who receive RT.

## 1 INTRODUCTION

The incidence rates of rectal cancer have been increasing, and the risk of the disease is shifting to younger populations gradually.^1^ Due to improvements in medical care, more rectal cancer patients are surviving and experiencing the sequelae of cancer treatment.^2^

Radiotherapy (RT), chemotherapy (C/T), and surgical resection are important treatments for rectal cancer patients. Acute side effects after treatment, such as gastrointestinal and genitourinary toxicities, have been discussed thoroughly in previous studies.^3^ However, few studies have discussed late side effects of pelvic irradiation.^4^

Pelvic fracture may be one of the late side effects of pelvic irradiation.^5^, ^6^ Approximately 50% of previously ambulatory women become confined to bed after a pelvic fracture,^7^ and pelvic fracture significantly increases mortality by 12%‐20% in the first year of follow‐up.^8^ The importance of early detection and prevention of pelvic fracture should be emphasized.

Most studies have discussed pelvic fracture after irradiation for gynecological and prostate cancers.^5^, ^9^ Very few studies have discussed pelvic fractures in rectal cancer patients.^4^, ^10^ This study aimed to evaluate the pelvic fracture risk of rectal cancer patients who received RT.

## 2 METHODS

### 2.1 Data source

We used Taiwan's National Health Insurance Database (NHIRD) as our data source. Taiwan's National Health Insurance was founded in 1995 via the Taiwanese government. More than 99% of Taiwanese are enrolled in this national insurance and receive its comprehensive medical care. Taiwan's NHIRD collects nationwide medical information in detail, such as inpatient and outpatient diagnoses, medical procedures, drug prescriptions, medical treatment duration, and medical costs. The specialists in the Registry of Catastrophic Illness Database (RCID), a subpart of the NHIRD, review the records of those who are newly diagnosed as cancer patients by reviewing medical records and pathological tissue confirmation. Patients who pass the peer review and receive a Catastrophic Illness Card will have better social benefits and financial support by Taiwan's government. This study was approved by the Institutional Review Board of Taipei Veterans General Hospital (2016‐05‐007BC).

### 2.2 Cohort selection

The cohort was composed of patients aged 20 years or older who were diagnosed as having a first primary rectal cancer (ICD‐9‐CM 154.0 and 154.1) from the NHIRD between 1 January 1996 and 31 December 2011, including inpatient and outpatient information. We enrolled rectal cancer patients who had received radical rectal surgery, such as abdominoperineal resection of the rectum, low anterior resection, local excision, transsacral rectosigmoidectomy, or posterior resection of the rectum. To decrease any interference from other treatments and patients with a poor prognosis, we excluded patients with a history of HIV infection, previous malignancy, and pathological fracture of any bone. In clinical practice, it is still difficult for doctors to differentiate from pathological fracture to insufficient fracture. Patients with pathological fracture may have poor prognosis and disease control,^11^ and those patients may also have higher chance of pathological fractures risk over other part of the body, including pelvis. Therefore, we excluded patients who already have diagnosis of pathological fracture of any place to decrease the potential interference. Because the study entry point is half year after radical rectal surgery, we excluded patients whose observation interval was less than 6 months after curative rectal surgery (before our study entry point starts).

We collected RT information from our cohort. Preoperative and postoperative RT in this study are defined as patients received RT within 6 months before and after radical rectal surgery, respectively. The total portal numbers for the entire RT course were recorded completely in the NHIRD. We defined our long‐ and short‐course RT using portal numbers (radiation portal number per fraction × fraction numbers), which has been used in previous studies.^12^, ^13^ The typical radiation regimen for preoperative long‐course RT and postoperative RT is 45‐50.4 Gy in 25‐28 fractions (usually more than three portals per day). Therefore, we included patients who had received more than 75 (3 × 25) portals within 6 months before and after radical rectal surgery as the long‐course RT group. Similarly, the typical radiation regimen for preoperative short‐course RT is 25 Gy in five fractions (usually four portals per day). Thus, we included patients who had received 18‐22 portals (approximately 20 portals (4 × 5)) within 6 months before radical rectal surgery as the short‐course RT group. In this study, we want to evaluate pelvic radiotherapy directly related to rectal cancer (such as neoadjuvant radiotherapy and postoperative pelvic radiotherapy). Therefore, patients who received miscellaneous RT portals, with a portal number not in the range of short‐course or long‐course definition, were excluded, which radiotherapy may relate to other reasons or palliative intention. The non‐RT group comprised patients who underwent radical rectal surgery but never received any RT portals.

The follow‐up time for each patient is ended on the date of diagnosis of any pelvic fracture, death, or the end of the study (31 December 2011), whichever occurred first.

Pelvic fracture is defined as patients with International Classification of Diseases, Ninth Revision, Clinical Modification (ICD‐9‐CM) Codes 808, 805.4‐805.7, 806.4‐806.7, and 820 (including pelvic, sacrum, lumbar vertebral fracture, and femoral neck fracture) during the follow‐up time.^14^, ^15^, ^16^, ^17^ The radiation field of rectal cancer is usually designed to cover tumor or tumor bed, mesorectal, presacral, and internal iliac bones, and sometimes also included external iliac nodes in some cases.^18^ In contrast to pelvic bone, arm would not be irradiated and can be used as a comparison to evaluate the effect of radiation. This kind of study design has been used in previous study.^19^ Arm fracture was defined as patients with ICD‐9‐CM Codes with 812, 813, and 814 (including fractures of the humerus, radius or ulna, and carpal bones) during the follow‐up.^17^

### 2.3 Treatment factors and comorbidities

The C/T agents were classified and collected by their Anatomical Therapeutic Chemical (ATC) code. Other demographic data such as age at diagnosis, sex, and comorbidities, including autoimmune diseases, chronic obstructive pulmonary disease, diabetes mellitus, dyslipidemia, end‐stage renal disease, liver cirrhosis, and hypertension were also collected from the NHIRD. The Charlson comorbidity index score for each patient was calculated according to each patient's comorbidities. Osteoporosis was defined as patients with ICD‐9 Codes 733.00‐733.03 and 733.9 diagnosed before the radical rectal surgery.

### 2.4 Statistical methods

We analyzed the pelvic fracture incidence of the whole cohort and evaluated other demographic features, including age, sex, Charlson comorbidity index score, osteoporosis, and C/T, comparing the RT and non‐RT groups. Pelvic fracture risk between short‐course and long‐course RT were analyzed and compared.

To reduce the potential influence of confounding variables, we used a propensity score matching. Propensity scores for RT for each patient were calculated using age, sex, Charlson comorbidity index score, and C/T. One‐to‐one exact matching using propensity scores, age, and sex were performed to classify them into an RT group and a non‐RT group. We compared the pelvic fracture incidence rate between the RT and non‐RT group, using person‐years as the denominator.

We used univariable and multivariable Cox regression to identify risk factors for pelvic fracture, including age, sex, Charlson comorbidity index score, RT, C/T, and osteoporosis. The same analysis was repeated for arm fracture. To further explore the time‐varying effect of RT, we calculated the hazard ratio and *P* value of each factor in multivariable Cox model in matched cohort with RT divided into 2‐year interval. Cox regression with age as the time scale (adjusted for follow‐up time) was also calculated. Subgroup analyses were used to assess the relative risk of pelvic fracture across possible risk factors. Interaction examinations of RT and each subgroup factors were performed using multivariable Cox models. We also calculated the hazard ratio of pelvic fracture in the RT group every 2 years after RT during the 10‐year follow‐up. The time‐varying hazard ratios were estimated, which means that the hazard ratios would be modeled as step functions, that is, different coefficients over different time intervals.^20^

The data processing was performed with Microsoft SQL Server 2012 (Microsoft Corp., Redmond, WA). All analyses were computed in R (version R‐3.4.3; http://www.r-project.org). A two‐sided *P* value less than 0.05 was considered statistically significant. The cox.zph function shipped with the *survival* package in R was used to examine the correlation between Schoenfeld residuals and time.^21^

## 3 RESULTS

### 3.1 Population demographics

**Figure 1:**
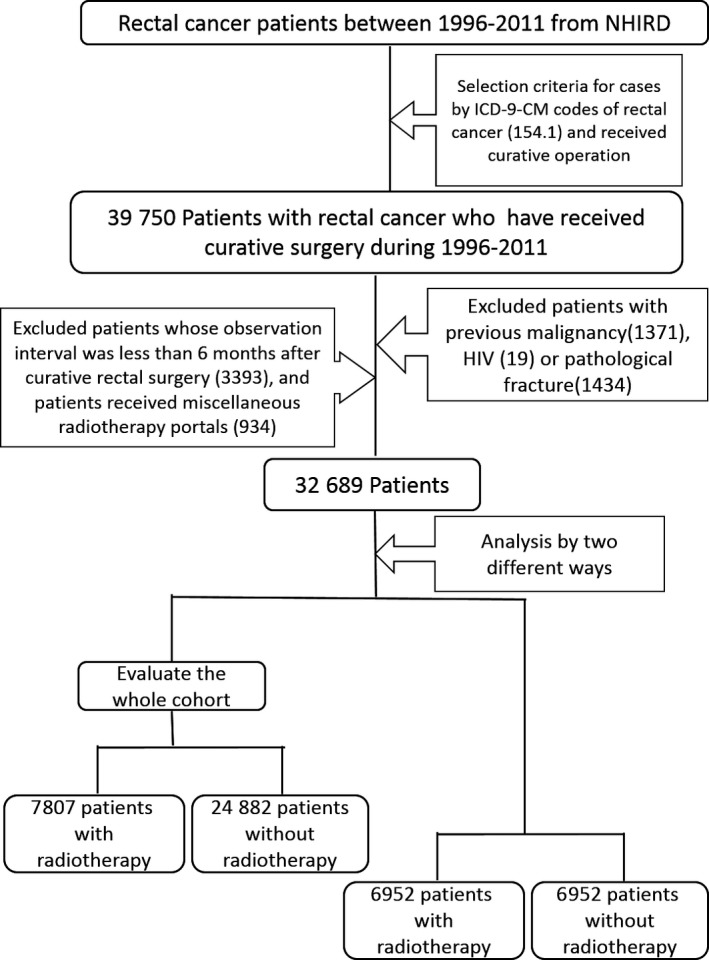
Research Flowchart (NHIRD: National Health Insurance Research Database)

We collected the records of 39 750 rectal cancer patients from NHIRD who had received curative rectal surgery from 1996 to 2011. After excluding patients with previous malignancies (1371 patients), patients with pathological fractures (1434 patients), patients with HIV infection (19 patients), patients whose observation interval was less than 6 months after curative rectal surgery (before our study entry point starts) (3393 patients) and patients receiving miscellaneous RT portal regiments (934 patients), 32 689 patients were selected (Figure 1).

Of the 32 689 patients who we studied, 7807 patients (23.9%) received RT, and 24 882 patients (76.1%) did not. There were 1616 patients who suffered from a pelvic fracture, an incidence of 1.17/100 person‐years among the whole cohort. Most patients received long‐course RT (6679 patients; 85.6%).

Before matching, we noticed that the distributions of the patient's sex and age were significantly different between patients who had received RT and those who did not. The RT group was younger than the non‐RT group (mean age 60.96 vs 65.06 years old, *P* < 0.001). The sex distribution was also significantly different. The male to female ratio was 63.1% to 36.9% in the RT group but 58.3% to 41.7% in the non‐RT group (*P* < 0.001). Therefore, propensity scores, age, and sex were used to perform one‐to‐one matches between the RT group and non‐RT group. After matching, 6952 patients in the RT group and 6952 patients in the non‐RT group were selected.

The median follow‐up time for all patients was 3.18 years (range from 1 day to 14.7 years). The percentages according to sex, osteoporosis diagnosis, C/T, average age at diagnosis, and the Charlson comorbidity index score of the cohort and matched populations were also recorded (Table 1).

**Table 1:** Patient characteristics

Factors	RT (Cohort) (N = 7807)	Non‐RT (Cohort) (N = 24882)	*P* value	RT (matched) (N = 6952)	Non‐RT (matched) (N = 6952)	*P* value
Age			<0.001			1.000
Mean (years)	60.96	65.06		61.83	61.83	
SD	12.50	12.52		11.70	11.70	
Range	8‐89	16‐91		24‐94	24‐94	
Sex			<0.001			1.000
Male	4923 (63.1%)	14494 (58.3%)		4352 (62.6%)	4352 (62.6%)	
Female	2884 (36.9%)	10388 (41.7%)		2600 (37.4%)	2600 (37.4%)	
Charlson Comorbidity Index score			0.108			1.000
Mean	6.47	6.42		6.56	6.56	
SD	2.59	2.72		2.48	2.48	
Range	2‐16	0‐19		2‐15	2‐15	
Osteoporosis			<0.001			0.003
Yes	3138 (12.6%)	860 (11.0%)		789 (11.3%)	679 (9.8%)	
No	21744 (87.4%)	6947 (89.0%)		6163 (88.7%)	6273 (90.2%)	
Chemotherapy			<0.001			<0.001
Yes	7206 (92.3%)	13091 (52.6%)		6367 (91.6%)	5405 (77.7%)	
No	601 (7.7%)	11791 (47.4)		585 (8.4%)	1547 (22.3%)	

RT, Radiotherapy; SD, standard deviation.

### 3.2 Pelvic fracture and arm facture results

After matching, there were 241 patients who suffered from pelvic fracture over 23 747.45 person‐years in the RT group (incidence rate: 1.01/100 person‐year), while 252 patients suffered from pelvic fractures over 30 609.78 person‐years in the non‐RT group (incidence rate: 0.82/100 person‐year). The pelvic fracture incidence was significantly higher in the RT group than in the non‐RT group (*P* = 0.020).

In multivariable Cox regression, RT was associated with an increased risk of pelvic fracture (HR: 1.246, 95% confidence interval (CI): 1.037‐1.495, *P* = 0.019), but it was not associated with an increased risk of arm fracture (HR: 1.013, 95% CI: 0.814‐1.259, *P* = 0.911). Older age (≥60 years old), female sex, osteoporosis, and a high Charlson comorbidity index score was correlated with an increased risk of pelvic fracture. The HR and *P* values of each factor in the univariable and multivariable Cox models are summarized in Table 2. Cox regression with age as the time scale (adjusted for follow‐up time) was calculated, which showed similar result, and was provided in the Table S2.

**Table 2:** The hazard ratio and *P* value of each factor in single variate and multivariable Cox model in matched cohort

	Univariable analysis		Multivariable analysis	
	Pelvic fracture	Arm fracture	Pelvic fracture	Arm fracture
	HR (95% CI); *P* value		HR (95% CI); *P* value	
Radiotherapy	1.209 (1.013‐1.444); 0.036	1.018 (0.824‐1.257); 0.87	1.246 (1.037‐1.495); 0.019	1.013 (0.814‐1.259); 0.911
Osteoporosis	2.898 (2.339‐3.591); <0.001	1.893 (1.418‐2.527); <0.001	1.426 (1.130‐1.800); 0.003	1.125 (0.826‐1.533); 0.454
Chemotherapy	0.773 (0.626‐0.955); 0.017	0.975 (0.744‐1.276); 0.852	0.947 (0.759‐1.181); 0.629	1.031 (0.7746‐1.372); 0.834
Age (>=60 vs <60 years)	1.090 (1.079‐1.100); <0.001	1.024 (1.014‐1.034); <0.001	1.086 (1.076‐1.097); <0.001	1.024 (1.014‐1.034); 630 < 0.001
Sex (Male vs female)	0.513 (0.429‐ 0.613); <0.001	0.401 (0.324‐0.497); <0.001	0.473 (0.390‐0.573); <0.001	0.384 (0.306‐0.482); <0.001
Charlson comorbidity index score	1.152 (1.113‐1.193); <0.001	1.084 (1.040‐ 1.130); <0.001	1.085 (1.050‐1.122); <0.001	1.068 (1.024‐1.115); 0.002

HR, hazard ratio; CI, confidence interval.

**Figure 2:**
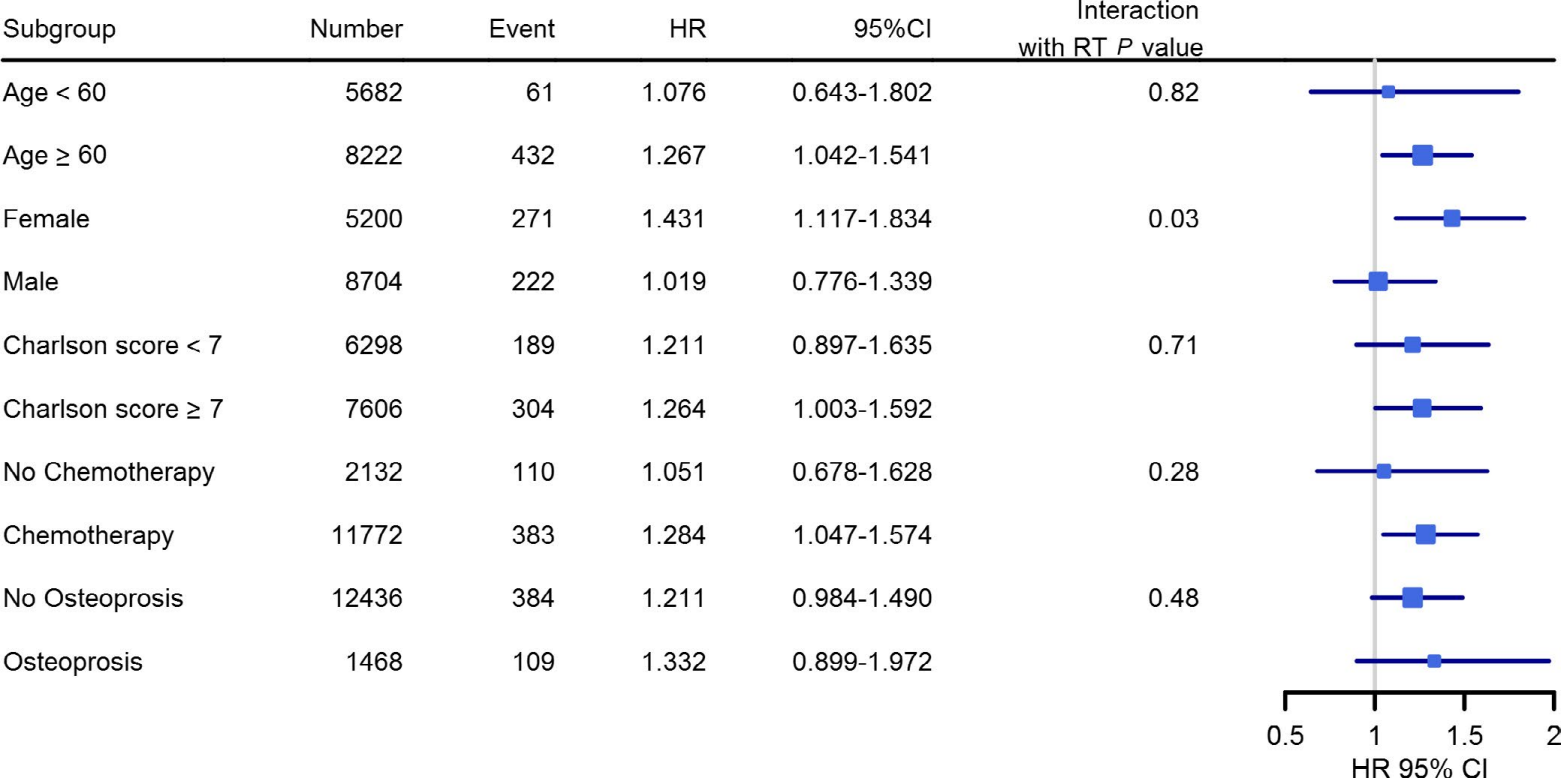
Subgroup analyses of different factors (age, sex, osteoporosis, chemotherapy, and Charlson comorbidity index score) of pelvic fracture risk in radiotherapy group and the *P* values of interactions with radiotherapy (HR, hazard ratio; CI, confidence interval)

To identify differences in pelvic fracture risk among the different categories of patients, subgroup analyses were performed. Subgroup analyses revealed that RT was associated with a higher risk of pelvic fracture in patients whose ages were over 60 years (HR: 1.267, 95% CI: 1.042‐1.541), those who received C/T (HR: 1.284, 95% CI: 1.042‐1.571), and those whose Charlson comorbidity index scores were more than 7 (HR: 1.264, 95% CI: 1.003‐1.592). Subgroup analyses also showed that RT was associated with a higher pelvic fracture risk in women (HR: 1.431, 95% CI: 1.117‐1.834) but not in men (HR: 1.019, 95% CI: 0.776‐1.339) (Figure 2). The *P* values of interaction examination with RT for each subgroup factor in the multivariable Cox model were shown in figure 2, and sex and RT had a significant interaction (*P *=* *0.03).

### 3.3 Other analysis results

**Figure 3:**
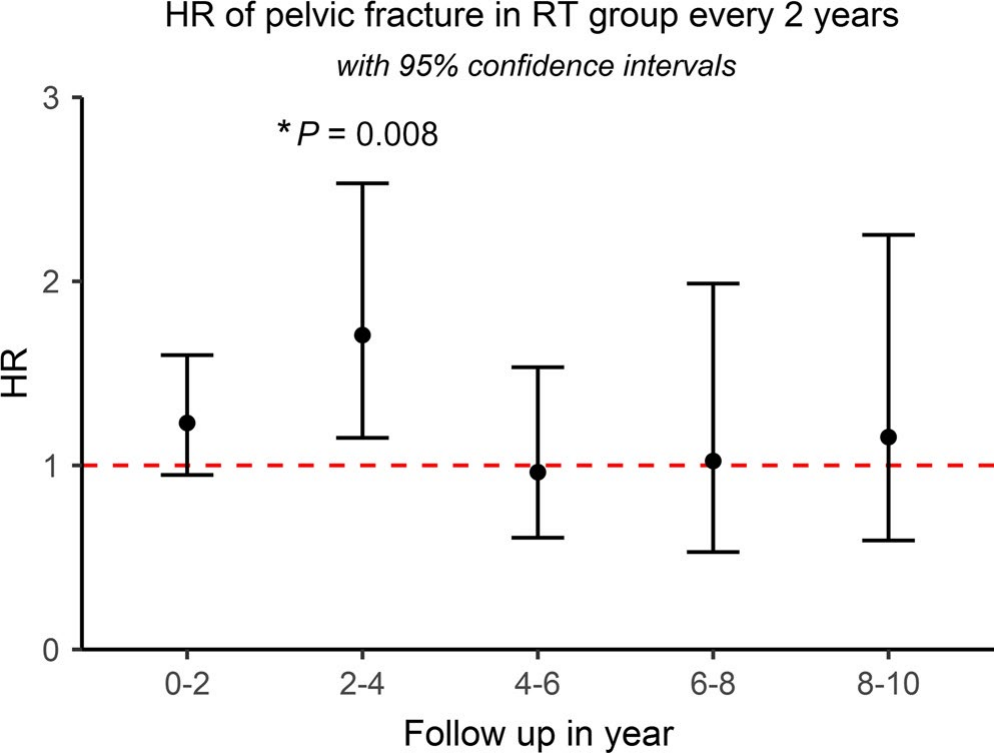
Hazard ratio of pelvic fracture in radiotherapy group every 2 years during 10 years of follow‐up. The hazard ratio of pelvic fracture risk is significantly increased during follow‐up 2‐4 years after radiotherapy (HR: 1.66, 95% CI:1.119‐2.468, *P* = 0.012) (HR, hazard ratio; CI, confidence interval)

In the multivariable Cox regression analysis of the cohort, there was no significant difference in pelvic fracture risk between long‐course and short‐course RT (*P *=* *0.972). We used a multivariable Cox model to evaluate the risk of pelvic fracture in the RT group every 2 years after RT during the 10 years of follow‐up. The hazard ratio of the pelvic fracture risk was significantly increased during follow‐up 2‐4 years after RT (HR: 1.707, 95% CI: 1.150‐2.534, *P *=* *0.008) (Figure 3). The pelvic fracture risk in the RT group during the first 2 years of follow‐up also tended to be higher, but the difference was not statistically significant (HR: 1.231, 95% CI: 0.948‐1.598, *P *=* *0.119). Further detailed HR and *P* value of each factor in multivariable Cox model in matched cohort with radiotherapy group divided into every 2 years during 10 years of follow‐up were provided in the Table S1. Proportional hazards assumption examination was done, and is not violated for radiotherapy (*P* = 0.745). Even if the assumption is violated for radiotherapy, the coefficient that we estimate for radiotherapy is a sort of average effect over the range of times observed in our study, which has been described in other reference.^22^

## 4 DISCUSSION

Post‐RT pelvic fracture has been discussed thoroughly for gynecological and prostate cancer,^9^, ^23^ but few studies have discussed it for rectal cancer.^4^ The survival rate of rectal cancer has been improved due to advances in treatment, and thus, late side effects among survivors have become extremely important.^3^ The morbidity and mortality risks after pelvic fracture are both high. It has been reported that 17%‐32% of all deaths are related to pelvic fractures,^24^ and more than half of patients cannot regain mobility within a year after the fracture.^25^ Pelvic fractures are also associated with infection, depression, and high medical costs.^26^, ^27^ It is a serious problem that needs to receive more attention. To our knowledge, this study has the largest patient population ever enrolled to evaluate pelvic fracture after irradiation among rectal cancer patients, with most previous studies only enrolling a few hundred patients or fewer.^2^, ^4^, ^28^

The mechanism through which RT causes fracture is as follows: radiation can reduce osteoblast numbers, arrest their cell cycles, cause apoptosis and result in perivascular damage.^5^ Preclinical data have shown that irradiation of the tibia of the rat results in osteoblast apoptosis and small trabeculae destruction and causes a 50% reduction in trabecular bone.^29^ A similar effect also occurs in humans, and in irradiated gynecological cancer patients, bone mineral density is lost by 11% over the L2 spine and 15.8% over the femoral neck after pelvic RT^30^; moreover, pelvic RT may cause cortical thinning of the femoral neck.^31^

Pain is the most common symptom of post‐RT pelvic fracture,^32^ although around 20%‐50% of people are asymptomatic.^33^ Pelvic insufficiency fracture (PIF) after radiation can be noted as quickly as 2 months or as long as 8 years after RT.^28^ The most common duration between treatment and fracture is approximately 6‐20 months.^32^, ^34^, ^35^ In our study, we noted that the pelvic fracture risk is higher after 2‐4 years of follow‐up after RT. The pelvic fracture risk in the RT group in the first 2 years of follow‐up tended to be higher but was not statistically significant. Therefore, we suggest that physicians pay more attention to pelvic fractures during the first 4 years of follow‐up among rectal cancer patients who receive pelvic RT. Surgery is an important treatment option for the post‐RT pelvic fracture, but most patients prefer to choose conservative treatments such as pain killers, analgesia, and bed rest.^5^, ^36^ Symptoms of pelvic fracture usually resolve after 1‐11 months of bed rest and conservative therapy.^37^

There are several factors discussed in previous studies that might increase the pelvic fracture rate among patients who received pelvic irradiation. Osteoporosis is one of the major risk factors for pelvic fracture. Patients with osteoporosis have a higher 5‐year PIF rate, approximately 15.6% compared with 2.9% for patients without osteoporosis (*P* = 0.01).^32^ Another study evaluated rectal cancer patients and reported that osteoporosis is a risk factor for sacral fracture (HR: 3.23, *P* = 0.02).^4^ Osteoporosis is also associated with a higher pelvic fracture rate in the multivariable Cox model in this study.

Whether C/T can result in a higher pelvic fracture rate or not is still uncertain. One study reported that the reduction rate in volumetric bone mineral density among patients who received C/T after 1 year was 15.9% over the L1‐L2 spine and 10.4% over the femoral neck.^30^ However, C/T was not a significant risk factor for pelvic fracture in several studies.^30^, ^32^, ^38^ Combining C/T with RT may cause a higher pelvic fracture rate based on a study of gynecology malignancy.^39^ In our study, C/T was a significant factor in single variable analysis but become insignificant after multivariable adjustment. In the subgroup analyses of the RT group, patients with C/T had a higher risk of pelvic fracture compared to the control.

Aging is also an important risk factor. A higher pelvic fracture risk after RT has been reported in many different studies in patients who are older than 50, 55, 60, 65, or 70.^36^, ^38^, ^40^, ^41^ Our study also proved that in a multivariable Cox model, patients older than 60 years had a higher pelvic fracture risk (HR: 1.086, CI: 1.076‐1.097, *P* < 0.001).

Women also had a significantly higher risk of pelvic fracture after RT. One study reported on 582 rectal patients and showed that women had a higher risk of sacral fracture after chemoradiation (HR: 2.64, *P* = 0.008).^4^ Another study reviewed 284 anal cancer patients and reported that all 4 patients experiencing pelvic fractures were women.^41^ Our study showed that women were at significantly higher risk for a pelvic fracture than men in the multivariable analysis (*P* < 0.001). In addition, we found that RT exposure was a significant risk factor in women but not in men. In the International Commission on Radiological Protection Publication (ICRP) in 2007, after a similar dose of radiation exposure, women have a higher risk of cancer and death than men.^42^ One study reported that women and men differ significantly in radiosensitivity (*P* = 0.004), which is associated with variations in single nucleotide polymorphisms.^43^ A group from Germany reported that estradiol treatment can increase the intrinsic radiosensitivity of breast cancer cells.^44^ Women generally have higher osteoporosis rates than men.^45^ Because of the lower baseline of bone health in women, the same degree of radiation–induced bone injury may have more of an impact on women than men. Currently, there is no sufficient evidence as to whether RT may cause more toxicity in women's bone health, and it is an important issue that needs additional studies.

In rectal cancer, recommended doses of radiation to the pelvis are typically 45‐50 Gy in 25‐28 fractions for long‐course RT^46^ and 25 Gy in five fractions for short‐course RT.^47^ There is only one study that directly compares the pelvic fracture risk between long‐course and short‐course RT,^48^ and there is no clear answer due to its limited patient number. One early study reported a relatively low fracture incidence of 2.6% in patients who received short‐course RT compared to long‐course RT, which showed a pelvic fracture cumulative incidence of approximately 3.1%‐7.1%.^4^, ^28^, ^49^ However, this result may have been affected by the different study design. In the current study, there was no significant difference in the pelvic fracture risk between the long‐course and short‐course RT groups (*P* = 0.972).

Thanks to improvements in the RT technique, the use of intensity‐modulated radiation therapy has been proven to reduce pelvic complications including pelvic fracture,^50^ and image‐guided radiation therapy can reduce the margins of the clinical target volume to planning target volume.^51^, ^52^ Using both techniques, the radiation volume and dose to the pelvic bones may be reduced and may reduce the risk of PIF in rectal cancer survivors.

There are several limitations of our study. First, the body mass index and lifestyle information of the patients are not available in the NHIRD, which makes it difficult to analyze those risk factors. Second, because we could not obtain the actual irradiation dose and volume via NHIRD, we could only use radiation portals as a surrogate of RT regimens. Third, we used the ICD‐9‐CM code to define osteoporosis and fracture events, which can sometimes underestimate the pelvic fracture risk if physicians do not record the proper diagnosis with an ICD‐9‐CM code.

In conclusion, an increased risk of pelvic fracture is noted in rectal cancer survivors, especially women, who receive RT.

## CONFLICT OF INTEREST

There is no funding disclosures in this article. There is no conflict of interest disclosures from any authors.
