# Supplementary material for: Increased risk of pelvic fracture after radiotherapy in rectal cancer survivors: A propensity matched study
Source: Cancer Med. 2019 May 18;8(8):3639–47. doi: 10.1002/cam4.2030 (PMC6639197; doi:10.1002/cam4.2030)
Supplement: Supplementary file 1 [file CAM4-8-3639-s001.docx]

**Supplement**

Supplement Table 1. The hazard ratio and p value of each factor in multi-variable Cox model in matched cohort with radiotherapy group divided into every 2 years during 10 years of follow-up

|  | **Multivariable analysis** |
| --- | --- |
|  | **Pelvic fracture** |
|  | **HR(95% CI);*p* value** |
| Radiotherapy |  |
| 0-2 years after RT | 1.230(0.948-1.560); 0.119 |
| 2-4 years after RT | 1.708(1.150-2.534); 0.008 |
| 4-6 years after RT | 0.963(0.606-1.532); 0.874 |
| 6-8 years after RT | 1.024(0.528-1.988); 0.944 |
| 8-10 years after RT | 1.154(0.591-2.253); 0.675 |
| Osteoporosis | 1.427(1.130-1.801); 0.003 |
| Chemotherapy | 0.947(0.759-1.181); 0.628 |
| Age (>=60 y vs. <60 y) | 1.086(1.075-1.097); <0.001 |
| Sex (Male vs. Female) | 0.474(0.391-0.574); <0.001 |
| Charlson Comorbidity Index score | 1.086(1.050-1.122); <0.001 |

HR: hazard ratio; CI: confidence interval

Supplement Table 2. The hazard ratio and p value of each factor in multi-variable Cox regression with age as the time scale (adjusted for follow-up time) in matched cohort

|  | **Pelvic fracture** | **Arm Fracture** |
| --- | --- | --- |
|  | **HR(95% CI);*p* value** | **HR(95% CI);*p* value** |
| Radiotherapy | 1.246 (1.040-1.494); 0.017 | 1.109(0.916-1.342);0.289 |
| Osteoporosis | 1.411 (1.119-1.780);0.004 | 1.267(1.000-1.604);0.050 |
| Chemotherapy | 0.951 (0.763-1.186); 0.657 | 0.859(0.685-1.077);0.187 |
| Sex (Male vs. Female) | 0.473 (0.390-0.573);<0.001 | 0.536(0.439-0.655);<0.001 |
| Charlson Comorbidity Index score | 1.085 (1.050-1.122);<0.001 | 1.073(1.037-1.111);<0.01 |
| Follow-up time  (per 2 years) | 0.851(0.791-0.917);<0.001 | 0.984(0.949-1.020);0.378 |

HR: hazard ratio; CI: confidence interval
